# Mr. Mortimer's Report of the Yellow or Bulam Fever

**Published:** 1817-02

**Authors:** 


### *Mr. Mortimer's Report of the Yellow or Bulam Fever.*

( Continued from page 17. )

THE history of the Regalia, a ship employed in the conveyance of troops from Guernsey to Sierra Leone; and from thence to Barbadoes with recruits for the black regiments there, may be deemed conclusive on this point, as shewing strongly the fatal effects of noxious vapours on the health of men, of whatever country or colour.

Although I had occasion to enter pretty largely into that history, in a letter addressed to the Commissioners, dated October 14, 1815; yet it being intimately connected with my present subject, I may be permitted to state the substance of it here, as follows:

"GENTLEMEN,

"I take leave to notice the arrival here of several transports from Sierra Leone with recruits for the black regiments on this station.

"The deplorable condition of these poor men on their debarkation; the unusual mortality during their passage, as well as since their coming to Barbadoes, have naturally excited a lively interest with the medical Gentlemen of the army, to discover, if possible, the causes leading thereto; none, however, sufficiently satisfactory, appear to have been adduced.

"Several hundred of these recruits had been embarked three weeks previously to their sailing out of the river of Sierra Leone, during which time their health was generally so good, as to render unnecessary the assistance of medicine. Their diet at sea was in every respect the same as whilst in the river, until the accession of sickness, when animal food was forbidden to those attacked.

"As the army medical officer, who had charge of the health of this detachment whilst at Sierra Leone, did not accompany them hither, we have no record of the period when disease first appeared, nor of the symptoms by which it was ushered in. The progress is learnt to have been rapid, and the termination almost invariably fatal.

"This mortality among those attacked, has experienced very little diminution at the Military Hospital, although the most active measures have been resorted to, with reference to the stage the disease had attained on admission.

"Dissection has exhibited the most convincing proofs of the fatal effects of high inflammatory action (when unsubdued by early depletion) in the general adhesions; in the various depositions, especially of water in the pericardium; whilst the enlarged state of the liver and mesenteric glands, as also the ulcerated intestine, may be further adduced in corroboration.

"In a disease, where the phænomena on dissection have been so pecnliarly striking, would it not be desirable to know what symptoms were most marked at the commencement? Whether there was previous indisposition? Whether the attack came on suddenly? If it was attended with much vascular action, heat of skin, or delirium? and if any of the latter, of what kind?

"These questions suggest themselves partly, because the surgeon of the Porcupine (which protected the transports until within sight of Barbadoes) was most assiduous in his attentions; and may, therefore, have it in his power to afford any information the Board may be pleased to require.

"I would attempt to describe the tout ensemble of the patient: It was bloated; the swelling unyielding and elastic; the breasts in particular more protuberant than the mammæ of a fat woman; the penis and scrotum partaking considerably of the general affection; the eye was inanimate; the mind listless; the appetite irregular and unnatural; the tongue white and moist; the pulse small and quick; urine in decreased quantity, and of a high colour.

"Whilst such were the appearances of some, there were others without these, recently attacked, suffering under the most acute feverish symptoms, and the highest degree of inflammatory action, accompanied by unusual restlessness, and pain on pressing the abdominal parietes. I saw some in this state, who, being largely bled, experienced considerable relief; and whose blood was firm, compact, and highly buffed.

"I should have to apologize for this apparently unnecessary, though faithful detail, were it not in some degree connected with the health of several of the white people belonging to the Regalia transport, and with the efforts I have deemed it a duty to make, since her return from Antigua to Carlisle Bay, to discover if possible the causes of its aberration.

"It may be well to premise, that the above ship left Guernsey in November 1814, for Plymouth, where she lay for seversl weeks with soldiers on board; from thence she proceeded first to Cork, and then to the African Coast, where she arrived on the 15th of February 1815, full of troops in perfect health. From this period to the latter end of June, she was employed in shifting troops and stores from one part to another. At this time she replenished her hold with water and wood, (the latter *cut down and brought on board the same day)* and received also more than a hundred black recruits to remain there until she sailed, which was three weeks afterwards.

"From her quitting Guernsey, up to within five days of this period, she had been perfectly healthy; neither her proper crew, nor any of the recruits had experienced any sickness.

"She at length quitted the river of Sierra Leone on the 18th of July for Barbadoes. A boy had been taken ill on the 13th, and died on the night of their departure. A few days after another boy was attacked, and died about the third day. The mate and carpenter were also seized on the passage, and died before the ship reached Barbadoes.

"A military officer and his lady, who had been some time on the coast, embarked in perfect health with the recruits; both were taken ill of the same fever, and both fell victims. Fourteen recruits were buried at sea.

"After arriving at Carlisle Bay, and whilst taking in water, the wife of the captain of the transport died of the fever; and, in a few days, Capt. Palmer himself followed.

"At the Saintes, a new mate was engaged in lieu of the one deceased; and it is worthy of observation, that he was taken out of another of the transports from the African Coast. He also died, (I believe) on the sixth day after being on board.

"On the Regalia being reported at Antigua to Commissioner Lewis, she was ordered to be fumigated, and to return to Barbadoes; within sight of this place, the only remaining mate, (then of necessity captain) fell ill; and anxious for his own life, quitted the ship and pulled in for the shore. It was well that he did; the symptoms were severe; and but for the early and copious abstraction of blood, it is probable, he would have followed the fate of his predecessors.

"As it was necessary there should be a superintendant on board during his absence, another mate was employed, who, on the sixth day was severely attacked; but as I happened to be on board at the time, he was immediately sent to the hospital, and by the same treatment recovered.

"Another man taken ill on the same day, was not admitted till twenty-four hours had passed away, and with them all chance of doing any service; disorganization of the stomach had taken place.

"With such a disease varying in its numbers of victims only with the decrease of the crew exposed to its attacks, which spread no farther than this ship, I lost no time in representing to the agent for transports my opinion of her condition, and of the propriety of taking every thing out of her hold, and exposing it to the certain effects of concentrated heat from stoves, the hatchways being closed.

"Experience had brought before me, on several occasions, the fatal influence of foul air on the health of a ship's company; the same had taught me to believe, that

(for the greater part) the devastating causes of fever are local; and as so many vessels were lying at anchor, alongside, with their crews perfectly free of disease, I could not otherwise than suspect the green wood to have had a baneful influence; it was stowed chock forwards, the spare cables abaft; and abaft those, small gravel ballast, looking extremely dirty.*

"After such pursuits as this ship had been engaged in from November to August, it would not be unreasonable to suppose, that amidst this mass much animal and vegetable matter had been commixed. That the seeds of disease existed in and were confined to this ship, has been made evident (I trust) by the previous history; and when I inform you, Sirs, that the Regalia was ordered to receive on board several hundred army invalids, to convey them first to Guadaloupe and thence to England, I feel every reliance on your approving of the steps I recommended to be taken by the agent for transports, especially when it is borne in recollection, that the ship's stay at Guadaloupe might be uncertain, where from particular causes, those sources of disease I have mentioned, would rather have received addition, than by any acts of cleanliness and attention on the part of the officers be diminished.

"I have the honour,

"&c. &c. &c.

"JOHN MORTIMER."

As respects the properties of noxious vapours, which indubitably existed in the Hankey in 1793, in the Nya-

---

* At many of the West India islands wood is extremely dear, and oftentimes not to be procured but with great difficulty.

To prevent those inconveniences, which would result from the detention of His Majesty's vessels from such a cause, the Commanders in Chief were in the habit of issuing a general order, that all ships requiring repair, either at Barbadoes, but particularly at Antigua, should previously to their arrival, complete a supply of wood and water for three months.

The exigencies of the service have frequently required unusual dispatch in the preparation and equipments; and it has thus not infrequently happened, that a vessel has proceeded to Dominica for the above purposes, when the wood has been cut down by the men of the ship and stowed away in the hold, already leaky, and thereby adding to the malign effects, which I believe to ensue from the *mere* decomposition of vegetable matter.

In such cases, I would account for the rise of fever in the Recruit, and of its influence on the working parties of the flag ship, after a greater or less period of exposure, according, perhaps, to the greater or less predisposition of the individual.

den, and in the Regalia, I think little doubt can be entertained of their malignant effects on the human frame, especially when with predisposition it is long exposed to their concentrated influence.

I can particularize many instances occurring during my nine years service in the West Indies, of men sent from the flag ship to expedite the equipment for sea of vessels in which fever has prevailed (in all probability from causes similar to those before dwelt on) who having been there attacked were received on board their own ship without, in any instance, communicating it to others.

On my quitting His Majesty's Ship Belleisle in 1808, I left her, as regarded acute diseases, perfectly healthy: very shortly after, working parties were sent on board the Recruit, where fever was common, and the major part of them partook of its influence after a few days occupation. The Pompeé, when commanded by Captain Cockburn, is another proof of the effect of miasmata in the crews of vessels.

Having captured to windward of Barbadoes, a French brig on her passage homewards from Martinique, whose men were suffering from the worst kind of fever, and where the effluvia from the hold were intolerable, almost every person sent on board was attacked. On the removal of the prisoners to the Pompeé, the malady prevailed very generally there; nor can it be wondered at, when we consider the very little attention paid to cleanliness by Frenchmen, and consequently the quantity of filth brought on board with their baggage. It is, moreover, worthy of remark, that disease continued to rage in the Prize until her hold was completely cleared, and exposed to the purifying effects of ventilation.

The many cases of fever which occurred in the Pompeé, after the removal of the prisoners into her (labouring under the malady) may at first sight give to it a character of infection, which it is no way entitled to.

The attacks were much more common among the prisoners, than with the crew; and those men first susceptible of it, and who chiefly suffered, were those whose duties placed them more particularly about the baggage.

Need it be wondered at, that in a vessel of French build, having the lower deck extremely low, (whilst the main was as strikingiy capacious) and arriving in a latitude where the thermometer ranges from seventy-eight to eighty-four; where, in addition to her proper crew, there were those of another man-of-war, and those again in a sickly state, and, therefore, taking up much additional room; that fever should continue until the removal of the diseased, and the free ventilation of the decks?

In the many hundred cases of fever placed under my care at Barbadoes, as well as at other hospitals, at different periods confided to me, I do not know of an instance of its communication to another patient under treatment at the same time for a different malady; and yet it may be presumed, that if any such instances had occurred in so long a period of seven years hospital practice (three of which were marked by the prevalence and fatality of this disease) I could hardly have failed to notice them.

It may be proper to observe, that the only precautions used here (the same I believe as at all other public establishments) are the ablution of the patient's body, and the removal of his linen to the wash house.

As regards liability to a second attack of fever, I may adduce the fatal case of Lieut. Reddie, of His Majesty's ship Gloire, who died at this hospital in 1809.

It would be satisfactory to have related fully the history of the previous fever, to which the second attack very quickly succeeded. But owing to the recent decease of the surgeon (Doctor M'Cormick) on the fifteenth day after his arriving in the country, and on the third of his seizure, and the subsequent dangerous illness of his successor, Mr. Richard Jones, (now surgeon to the Establishment at Bermuda) the Report was not regularly kept up. I can therefore only observe, that being called on during Mr. Jones's illness, I conceived Mr. Reddie to have the same kind of disease of which the Doctor had died, and with which Mr. Jones and Lieut. Curran (now Post Captain) were struggling.

Mr. Reddie being recovered, had dined at Bridgetown, and after his return was attacked in the night with fever, which terminated fatally.

The following note on the subject, was inserted in the hospital case book, dated Monday, November 14, 1809.

"Lieutenant Reddie died this morning at half after eleven, having been vomiting, and passing per anum, a brown, bloody looking fluid, several hours previously."

I saw Mr. Reddie on Monday evening last, for the first time; he had been a patient before for the case of fever, attended with considerable alvine obstructions. This state was present on my first visit; and not until a variety of cathartics, aided by the warm baths, &c. had been actively continued, was the belly freed of its load. Fever increased, and along with it, irritability of stomach, which continued to the last.

The effervescent draughts; fixed air in other forms; æther; and pepper, were, in conjunction and separately, administered without apparent effect; and with as little success were blisters applied to the head and stomach.*

During my short residence at Mariegalante, several cases of second seizure came under my observation. I have known men, who, happy in having once escaped the dangers of the malady, and confident of future exemption, have, while holding out such hope to their suffering brethren, been again invaded; and after a struggle, more or less protracted, have fallen a sacrifice to its rage.

Others, less assured of their safety, have sought it in the higher lands; and in the most diversified situations have exemplified the truth, that there is no positive security from the recurrence of this disease.

There are now occurring at this island (the topography of which has been so often described by medical writers, as peculiarly favourable to health) cases of fever in form most dissimilar, but in termination alike fatal. While in some, the early symptoms were so insidious and obscure, as to deceive the most wary practitioner, insomuch, that as late as the fifth day, the first indication of danger was discoverable in the commencing irritability of stomach, and the total suspension of the urinary secretion; in others, the high febrile action from the commencement, added to that anxiety of countenance, invariably attendant on the worst form of the disease, have warned the physician of the inefficiency of his art; but, in every case, black vomit closed the scene.

When I stated that this fever attacked in the month of December, natives, advanced in years, of the most retired and abstemious habits, and that it was not in any instance communicated to one of their anxious attendants, will not these facts be considered corroborative of the opinion, that to localities, and not to any infectious properties, ought this malady to be attributed?

I have shewn, that while it prevailed generally, and in full malignity all over the island of Mariegalante, where there were troops posted, the squadron at anchor close off the town, remained in the enjoyment of perfect health, al-

* The force of this relation of "second attacks" is considered by some, as much weakened by the quickness with which they appear to have succeeded the first: so as to deserve rather the term "relapse," than second seizure. But the intermission was perfect; the absence of all febrile affection of some days duration; and the strength sufficiently recruited to admit a journey of two miles on foot in a tropical climate.

though the communication was constant, being never interdicted. I have also shewn, that when any urgency of occasion has called for extraordinary exertion to refit vessels in which fever prevailed, the working parties sent on board have sometimes been seized, without ever communicating to their own ship's company on their return, the fever they have brought.

Had that at Mariegalante been infectious, we might have expected to find some of the inhabitants partaking of its influence; instead of being affected with intermittents, at the time, when great heat succeeding to heavy rain, produced in the newly arrived European, continued fever of a most malignant nature. And here I may notice, that the effects of intemperance were strongly exemplified in the more early attack of its votaries, as well as in the greater degree of danger.

From what has been advanced, it may be inferred, that the scourge of the West Indies, by some called "Bulam," by others, "ardent," but commonly "yellow" fever, is not a disease "sui generis," nor communicable from one person to another, in any of its forms or stages.

I am of opinion, that vapours emitted from the commixture of animal and vegetable substances, in a state of decomposition, are frequent sources of the worst kind of yellow fever in hot climates. I am supported in this opinion, by the numerous examples happening in my own practice (a few of which have been related) and by others occurriug to men of unquestioned veracity, as well as indefatigable research.

( To be continued. )

